# Severe disturbance of glucose metabolism in peripheral blood mononuclear cells of schizophrenia patients: a targeted metabolomic study

**DOI:** 10.1186/s12967-015-0540-y

**Published:** 2015-07-14

**Authors:** Mei-Ling Liu, Xiao-Tong Zhang, Xiang-Yu Du, Zheng Fang, Zhao Liu, Yi Xu, Peng Zheng, Xue-Jiao Xu, Peng-Fei Cheng, Ting Huang, Shun-Jie Bai, Li-Bo Zhao, Zhi-Guo Qi, Wei-Hua Shao, Peng Xie

**Affiliations:** Department of Neurology, The First Affiliated Hospital of Chongqing Medical University, 1 Youyi Road, Yuzhong District, Chongqing, 400016 People’s Republic of China; Institute of Neuroscience and the Collaborative Innovation Center for Brain Science, Chongqing Medical University, Chongqing, China; Chongqing Key Laboratory of Neurobiology, Chongqing, China; Department of Neurology, The Third People’s Hospital of Chongqing, Chongqing, China; Department of Respiratory Medicine, The First Affiliated Hospital of Chongqing Medical University, Chongqing, China

**Keywords:** Schizophrenia, Major depression, Glucose metabolism, GC–MS

## Abstract

**Background:**

Schizophrenia is a widespread and debilitating mental disorder. However, the underlying molecular mechanism of schizophrenia remains largely unknown and no objective laboratory tests are available to diagnose this disorder. The aim of the present study was to characterize the alternations of glucose metabolites and identify potential diagnostic biomarkers for schizophrenia.

**Methods:**

Gas chromatography/mass spectrometry based targeted metabolomic method was used to quantify the levels of 13 glucose metabolites in peripheral blood mononuclear cells (PBMCs) derived from healthy controls, schizophrenia and major depression subjects (n = 55 for each group).

**Results:**

The majority (84.6%) of glucose metabolites were significantly disturbed in schizophrenia subjects, while only two (15.4%) glucose metabolites were differently expressed in depression subjects relative to healthy controls in both training set (n = 35/group) and test set (n = 20/group). Antipsychotics had only a subtle effect on glucose metabolism pathway. Moreover, ribose 5-phosphate in PBMCs showed a high diagnostic performance for first-episode drug-naïve schizophrenia subjects.

**Conclusion:**

These findings suggested disturbance of glucose metabolism may be implicated in onset of schizophrenia and could aid in development of diagnostic tool for this disorder.

**Electronic supplementary material:**

The online version of this article (doi:10.1186/s12967-015-0540-y) contains supplementary material, which is available to authorized users.

## Background

Schizophrenia is a multifaceted and devastating mental disorder affecting up to 1% of the general population [1]. It causes progressive functional decline, lifelong disability and tremendous socioeconomic burden for patients [2, 3]. Despite extensive researches into schizophrenia, the definite pathogenesis of schizophrenia remains largely unknown. Currently, diagnosis of schizophrenia primarily relies on subjective identification of symptom clusters by psychiatrist. However, this subjective diagnostic modality results in a considerable error rate due to the complex spectrum of symptoms and their similarities to other mental disorders [4]. In light of these facts, it is greatly needed to explore the molecular mechanism and develop objective diagnostic biomarkers for schizophrenia.

Metabolites represent the final product of interactions among various factors including genetic, physiological and environmental factors, and may better reflect the functional status of individuals. Quantifiable differences in metabolite patterns provide valuable clues to uncover the mechanism and develop diagnostic biomarkers for psychiatry disorders [5, 6]. Recently, our group has characterized metabolite signatures associated with stress resilience and suicide [7, 8]. Besides, we have identified diagnostic metabolite biomarkers for major depression and bipolar disorder [9, 10].

As to schizophrenia, early studies have identified disturbances of several metabolic pathways, such as oxidative stress, lipid and amino acid metabolism [11–13]. Specially, it was repeatedly reported that schizophrenia patients showed disturbance of several glucose metabolite intermediates in periphery and brain [14–16]. Transcriptomics and proteomics studies also displayed abnormal expression of glucose metabolism-related enzymes [17, 18]. Moreover, glucose regulation is related to verbal declarative memory performance in schizophrenia patients [19]. These scattered evidences indicated glucose metabolic pathway may be disturbed in schizophrenia. However, currently there has not been a comprehensive global evaluation of glucose metabolic pathway in schizophrenia. More importantly, it remains unclear whether and how specific changes of glucose metabolism might account for schizophrenia.

Brain tissues and cerebrospinal fluid seem to be the most suitable biological samples for schizophrenia research. However, brain tissue biopsy and lumbar puncture cannot be practically applied in schizophrenia patients due to ethical and safety constraints. In contrast, peripheral blood mononuclear cells (PBMCs) can be easily collected at minimal risk and cost and allow to capture the freshly isolated systemic cellular reactivity in the early phases of the disorder [20]. Besides, PBMCs may reflect molecular processes in the central nervous system as the brain and PBMCs show a number of parallel responses [21–23]. Moreover, peripheral immune dysfunction was widely implicated in the pathogenesis of schizophrenia [24, 25]. Significantly, previous studies have indicated that such cells are useful peripheral sources to identify the underlying pathogenic mechanisms and diagnostic markers for other psychiatric disorders such as autism, depression and borderline personality disorder [26–28]. Considering the above information, PBMCs were thus chosen as the sample source in this study.

Here, using a gas chromatography/mass spectrometry (GC/MS) based targeted metabolomic method, we simultaneously quantified the levels of 13 glucose metabolites in PBMCs derived from schizophrenia, major depression and healthy control groups (n = 55 for each group). Our aim was to characterize how the glucose metabolites change in the early stage of schizophrenia, and identify the potential diagnostic PBMC metabolite biomarkers for schizophrenia. Besides, we sought to determine whether antipsychotic drugs effect on glucose metabolism pathway.

## Methods

### Ethics statement

Prior to sample collection, written informed consents were obtained from all recruited subjects. The protocols of this study were reviewed and approved by the Ethical Committee of Chongqing Medical University. All procedures were conducted according to the principles expressed in the Declaration of Helsinki.

### Participants

Totally, 55 schizophrenia and 55 major depression subjects were recruited from the psychiatric center in the First Affiliated Hospital of Chongqing Medical University. All diagnoses were performed according to the Structured Psychiatric Interview using the DSM-IV-TR criteria [29]. Candidates with pre-existing physical or other mental disorders, or illicit drug use were excluded. During the same time period, 55 healthy controls were enrolled from the medical examination center in the First Affiliated Hospital of Chongqing Medical University. Healthy controls were required to have no current or previous lifetime history of neurological, DSM-IV Axis I/II, or systemic medical illness.

The recruited samples were divided into a training set and a test set. The training set, comprising healthy controls, first-episode drug-naïve schizophrenia and major depression subjects (n = 35/group), was used to characterize the unique glucose metabolic pattern and identify the potential diagnostic markers for schizophrenia. The test set, comprising 20 schizophrenia subjects, 20 major depression subjects and 20 healthy controls, was used to independently validate the identified glucose metabolic pattern of schizophrenia and assess the effect of antipsychotics on glucose metabolites.

### Sample preparation and GC/MS acquisition

Fasting blood samples were collected into EDTA-coated tubes and were centrifuged at 3,000 rpm for 15 min at 4°C to obtain plasma. Subsequently plasm was overlaid onto Ficoll-Paque Plus (GE Healthcare Bio-sciences AB, Sweden), and centrifuged at 2,000 rpm for 20 min at room temperature. The purified PBMCs were harvested and washed three times in phosphate buffer saline. After that, each PBMC sample was divided into equal aliquots, transferred into liquid nitrogen for 3 min and then stored at −80°C until undergoing later analysis.

For GC–MS analysis, each aliquot of PBMCs, which was isolated from 5 ml blood sample, was added with a 400 μL solution of water–methanol–chloroform (2:5:2, v/v/v). After vortexing for 30 s and standing overnight at −20°C, the mixture was sonicated for 3 min and subsequently centrifuged at 14,000*g* for 10 min at 4°C. 300 μL supernatant was collected, and the residue was resuspended in 200 μL methanol. The residue was then vortexed and centrifugated as before. 200 μL supernatant of the residue was extracted and mixed with the first. A 250 μL aliquot of mixed supernatant was evaporated to dryness under a stream of nitrogen gas. The dried metabolic extract was derivatized first with 30 µL of methoxamine (20 mg/mL) for 90 min at 37°C with continuous shaking. Subsequently, 30 µL of BSTFA with 1% TCMS was added to the mixture and heated for 1 h at 70°C to form trimethylsilyl (TMS) derivatives. After derivatization and cooling to room temperature, 1 μL of this derivative was injected in the GC/MS for analysis.

### GC/MS analysis

GC/MS analysis was carried out according to this group’s previously published work [7, 30]. Briefly, each 1 μL of the derived sample was injected into an Agilent 7890A GC system (Agilent Technologies Inc., USA). An HP-5 MS fused silica capillary column (30 m × 0.25 mm × 0.25 μm, Agilent, USA) was used for metabolite separation with helium carrier gas at a flow rate of 1 mL/min. The injector temperature was set at 280°C. The column temperature was initially kept at 80°C for 2 min and then increased to 320°C at 10°C/min, where it was held for 6 min. The column effluent was introduced into the ion source of an Agilent 5975 mass selective detector (Agilent Technologies). The MS quadrupole temperature was set at 150°C, and the ion source temperature was set at 230°C. Data acquisition was performed first in the full-scan mode (scanning range from 50 to 550 m/z) and then in selected ion monitoring (SIM) mode for quantification. The characteristic fragment ions and retention times of metabolites were shown in Additional file 1: Table S1. All samples were analyzed consecutively at random. A quality control (QC) sample, pooled from a representative PBMCs sample of each group, was added in each batch of analyses in order to adjust the variations between batch variability.

### Targeted metabolomic data analysis

Mass spectral data were converted to NetCDF format and then processed by XCMS software for peak finding, integration and alignment. The optimized XCMS parameters were set as follows: method = “matchedFilter”; full width at half maximum (fwhm) = 4.0; signal-to-noise cutoff (snthresh) = 10.0; retention time window (bw) = 3. Each metabolite concentration was expressed in relative abundance (metabolite peak area of study sample divided by that of QC sample) before the following statistical analysis.

### Identification of PBMC metabolite biomarkers for schizophrenia

As clinical diagnosis based on the quantification of a small number of metabolites would be more practical, a stepwise optimization algorithm based on Akaike’s information criterion (AIC) was applied to optimize the metabolite biomarker combination [9, 10]. To evaluate the diagnostic generalizability of the schizophrenia biomarker, the ability of the simplified biomarker panel to discriminate schizophrenia subjects from non-schizophrenia subjects was quantified using a receiver-operating characteristic (ROC) curve analysis [31].

### Statistical analysis

The Chi square test was applied to analyze categorical data (sex). All continuous variables such as age, BMI and metabolite concentrations were analyzed using one-way ANOVA followed by Bonferroni post hoc test. All continuous variables were expressed as means ± standard errors of the mean. The statistical analyses were carried out with SPSS software (version 17.0). A *p* value of less than 0.05 was considered to be statistically significant.

## Results

### Demographic and clinical characteristics

The detailed demographic and clinical characteristics of the recruited subjects are presented in Table 1. The demographic parameters such as age, gender and BMI were not distinguishable among these three groups in both training set and test set.

**Table 1:** Demographic characteristics of the recruited subjects

	Variables	SZ	MD	HC	Statistics	*P* ^a^
Training set	Sample size	35	35	35	–	–
	Medication (Y/N)	N	N	N	–	–
	Sex (M/F)	14/21	17/18	18/17	χ^2^ = 1.00	0.61
	Age (years)^b^	32.49 ± 14.12	36.40 ± 10.65	36.54 ± 5.98	F = 1.60	0.21
	BMI^b^	22.60 ± 1.95	22.09 ± 2.12	22.21 ± 1.72	F = 0.67	0.52
Test set	Sample size	20	20	20	–	–
	Medication (Y/N)	6/14	N	N	–	–
	Sex (M/F)	9/11	11/9	10/10	χ^2^ = 0.40	0.82
	Age (years)^b^	28.45 ± 2.09	27.55 ± 2.05	30.15 ± 1.65	F = 0.47	0.63
	BMI^b^	21.86 ± 0.50	21.70 ± 0.55	21.74 ± 0.48	F = 0.03	0.98

Chi square analyses for categorical variables (sex).

*SZ* schizophrenia, *MD* major depression, *HC* healthy controls, *M/F* male/female.

^a^ANOVA test for continuous variable (age, BMI).

^b^Values were expressed as mean ± SEM.

### Characterization of differentially expressed metabolites between schizophrenia subjects and healthy controls

**Figure 1:**
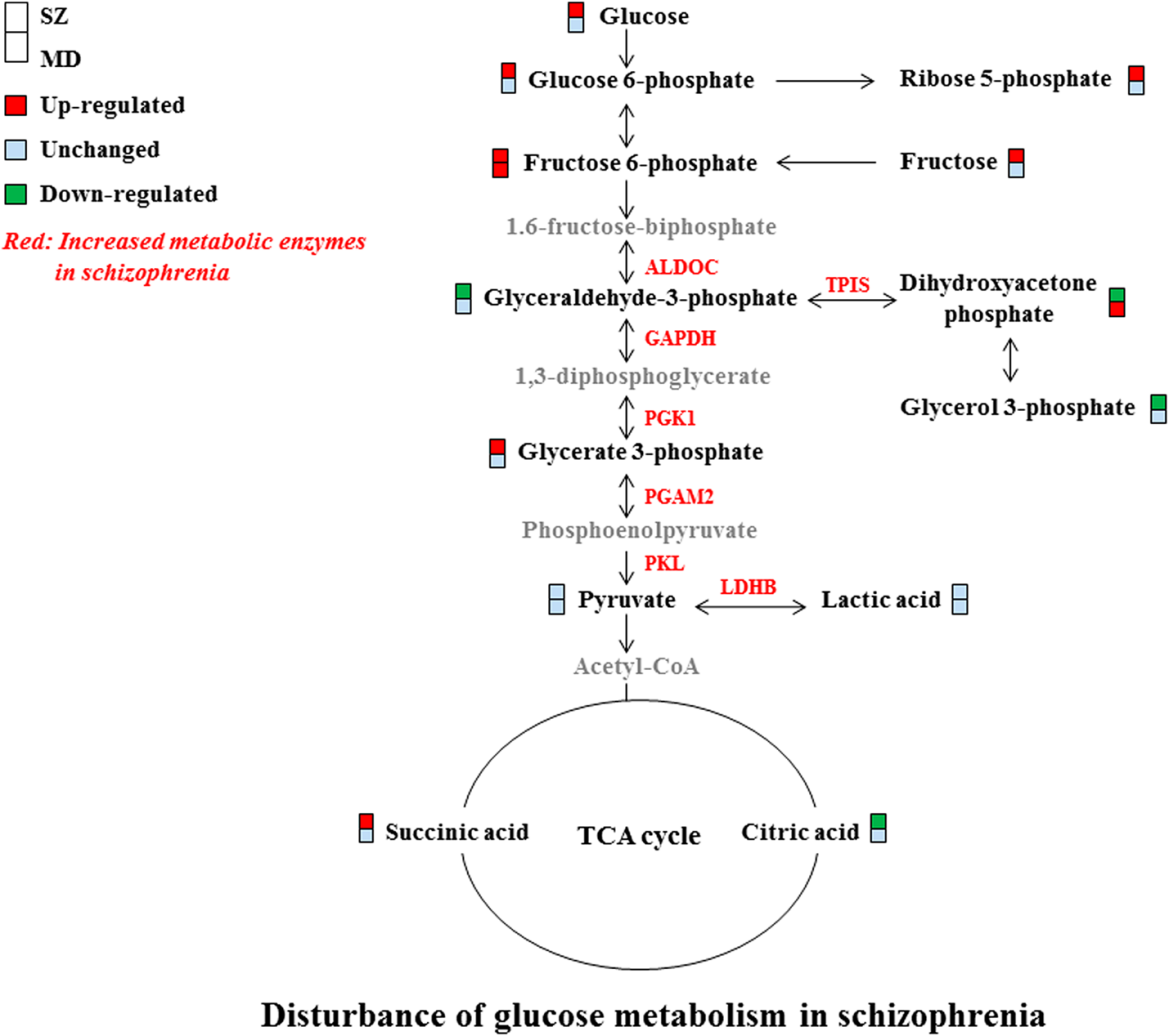
Differential metabolites and metabolic enzymes involved in glucose metabolism. All these metabolites were compared among three groups in training set. *Red-colored boxes* indicate increased metabolites in schizophrenia or major depression subjects compared to healthy controls, while *green-colored boxes* indicate decreased metabolites; *Red letters* indicate upregulated metabolic enzymes in PBMCs of schizophrenia subjects as previous proteomic study reported [18]. *ALDOC* aldolase C, *TPIS* triosephosphate isomerase, *GAPDH* glyceraldehydes-3-phosphate dehydrogenase, *PGK1* phosphoglycerate kinase 1, *PGAM2* phosphoglycerate mutase 2, *PKL* pyruvate kinase, *LDHB* lactate dehydrogenase B.

Using GC–MS approach, we measured 13 key metabolites of glucose metabolism pathways in PBMCs. In training set, 11 metabolites (84.6%) were differentially expressed in first-episode drug-naïve schizophrenia subjects relative to healthy controls (Table 2; Figure 1). Thereinto, seven metabolites were significantly increased in schizophrenia subjects relative to healthy controls, including glucose, glucose 6-phosphate, fructose, fructose 6-phosphate, glycerate 3-phosphate, succinic acid and ribose 5-phosphate. Moreover, four metabolites were significantly decreased in schizophrenia subjects relative to healthy controls, including glyceraldehyde-3-phosphate, dihydroxyacetone phosphate, glycerol 3-phosphate and citric acid. The remaining metabolites, pyruvate and lactic acid, did not differ between these two groups.

**Table 2:** The differentially expressed metabolites between SZ and HC group

Metabolites	Metabolic pathway	Training set		Test set	
		Log_2_(FC)^a^	*P* value^b^	Log_2_(FC)^a^	*P* value^b^
Glucose	Glycosis	4.22	**0.000**	0.67	**0.000**
Glucose 6-phosphate	Glycosis	1.84	**0.000**	1.48	**0.000**
Fructose 6-phosphate	Glycosis	1.84	**0.000**	1.06	**0.000**
Fructose	Glycosis	6.12	**0.000**	0.48	**0.003**
Glyceraldehyde-3-phosphate	Glycosis	−1.91	**0.000**	−1.05	**0.006**
Dihydroxyacetone phosphate	Glycosis	−2.28	**0.000**	−1.28	**0.000**
Glycerol 3-phosphate	Glycosis	−0.6	**0.000**	−0.49	**0.001**
Glycerate 3-phosphate	Glycosis	0.88	**0.000**	0.28	0.363
Pyruvate	Glycosis	0.54	0.090	0.82	**0.013**
Lactic acid	Glycosis	−0.11	1.000	−0.79	**0.002**
Citric acid	TCA	−1.08	**0.000**	−0.71	**0.001**
Succinic acid	TCA	3.08	**0.000**	0.76	**0.001**
Ribose 5-phosphate	Pentose phosphate pathway	4.53	**0.000**	−0.39	0.133

Values given in bold denote statistically significant results (*P* < 0.05).

*SZ* schizophrenia, *HC* healthy controls, *FC* fold change, *TCA* tricarboxylic acid cycle.

^a^A negative log_2_(FC) indicated significantly lower expression in schizophrenia subjects compared to healthy controls; a positive log_2_(FC) indicated significantly higher expression in schizophrenia subjects compared to healthy controls.

^b^These data were analyzed using one-way ANOVA followed by Bonferroni post hoc test between two groups of each cohort.

Additionally, the results aforementioned could be replicated in independent test set except for four metabolites, including glycerate 3-phosphate, pyruvate, lactic acid and ribose 5-phosphate (Table 2). In detail, compared to healthy controls, levels of glycerate 3-phosphate and ribose 5-phosphate in schizophrenia subjects were significantly higher in training set, but did not differ in test set. On the contrary, relative to healthy controls, levels of pyruvate and lactic acid in schizophrenia subjects were not distinguishable in training set, but were significantly differently expressed in test set.

To test whether antipsychotics influenced glucose metabolism, we compared levels of glucose metabolites between unmedicated and medicated schizophrenia subjects in test set (Additional file 1: Table S2). Of these 13 metabolites, only ribose 5-phosphate were differently expressed between unmedicated and medicated schizophrenia subjects. These findings demonstrated that antipsychotics may not significantly influence glucose metabolism in individuals with episode schizophrenia.

### Characterization of differentially expressed metabolites between depression subjects and healthy controls

In opposite to schizophrenia, only 2 of 13 (15.4%) glucose metabolites were differently expressed between depression subjects and healthy controls in both training set and test set (Table 3; Figure 1). The majority of results were consistent in both sets. Specifically, fructose 6-phosphate was significantly increased in depression subjects relative to healthy controls in training set, but were not distinguishable between these two groups in test set. Compared to healthy controls, dihydroxyacetone phosphate in depression subjects were significantly increased in training set, but decreased in test set. Moreover, compared to healthy controls, levels of succinic acid in depression subjects were not distinguishable in training set, but were significantly increased in test set.

**Table 3:** The differentially expressed metabolites between MD and HC group

Metabolites	Metabolic pathway	Training set		Test set	
		Log_2_(FC)^a^	*P* value^b^	Log_2_(FC)^a^	*P* value^b^
Glucose	Glycosis	0.50	1.000	0.34	0.239
Glucose 6-phosphate	Glycosis	0.74	0.228	0.38	1.000
Fructose 6-phosphate	Glycosis	0.99	**0.019**	0.18	1.000
Fructose	Glycosis	0.08	1.000	0.29	0.198
Glyceraldehyde-3-phosphate	Glycosis	−0.09	0.907	−0.25	0.802
Dihydroxyacetone phosphate	Glycosis	0.71	**0.000**	−0.79	**0.004**
Glycerol 3-phosphate	Glycosis	−0.09	1.000	−0.10	1.000
Glycerate 3-phosphate	Glycosis	−0.71	0.190	−0.28	0.692
Pyruvate	Glycosis	0.34	0.606	0.22	1.000
Lactic acid	Glycosis	−0.15	1.000	−0.31	0.239
Citric acid	TCA	−0.09	1.000	−0.11	1.000
Succinic acid	TCA	0.07	1.000	0.71	**0.003**
Ribose 5-phosphate	Pentose phosphate pathway	−0.03	1.000	−0.16	1.000

Values given in bold denote statistically significant results (*P* < 0.05).

*MD* major depression, *HC* healthy controls, *FC* fold change, *TCA* tricarboxylic acid cycle.

^a^A negative log_2_(FC) indicated significantly lower expression in major depressive subjects compared to healthy controls; a positive log_2_(FC) indicated significantly higher expression in major depressive subjects compared to healthy controls.

^b^These data were analyzed using one-way ANOVA followed by Bonferroni post hoc test between two groups of each cohort.

### Characterization of differentially expressed metabolites between schizophrenia and depression subjects

In training set, 11 of 13 glucose metabolites were differently expressed between schizophrenia and depression subjects (Table 4). Coincidentally, these 11 altered metabolites were also differently expressed between schizophrenia subjects and healthy controls in training set.

**Table 4:** The differentially expressed metabolites between SZ and MD group

Metabolites	Metabolic pathway	Training set		Test set	
		Log_2_(FC)^a^	*P* value^b^	Log_2_(FC)^a^	*P* value^b^
Glucose	Glycosis	3.73	**0.000**	0.32	0.071
Glucose 6-phosphate	Glycosis	1.09	**0.000**	1.10	**0.000**
Fructose 6-phosphate	Glycosis	0.85	**0.000**	0.88	**0.001**
Fructose	Glycosis	6.04	**0.000**	0.19	0.342
Glyceraldehyde-3-phosphate	Glycosis	−1.83	**0.000**	−0.80	0.114
Dihydroxyacetone phosphate	Glycosis	−2.99	**0.000**	−0.49	0.849
Glycerol 3-phosphate	Glycosis	−0.52	**0.000**	−0.39	**0.020**
Glycerate 3-phosphate	Glycosis	1.59	**0.000**	0.56	**0.022**
Pyruvate	Glycosis	0.2	1.000	0.60	0.057
Lactic acid	Glycosis	0.04	1.000	−0.48	0.198
Citric acid	TCA	−0.98	**0.000**	−0.60	**0.008**
Succinic acid	TCA	3.01	**0.000**	0.06	1.000
Ribose 5-phosphate	Pentose phosphate pathway	4.56	**0.000**	−0.23	0.820

Values given in bold denote statistically significant results (*P* < 0.05).

*SZ* schizophrenia, *MD* major depression, *FC* fold change, *TCA* tricarboxylic acid cycle.

^a^A negative log_2_(FC) indicated significantly lower expression in schizophrenia subjects compared to major depressive subjects; a positive log_2_(FC) indicated significantly higher expression in schizophrenia subjects compared to major depressive subjects.

^b^These data were analyzed using one-way ANOVA followed by Bonferroni post hoc test between two groups of each cohort.

In test set, only five metabolites were consistently differently expressed between schizophrenia and depression subjects (Table 4). However, the changes of other five metabolites (glucose, fructose, glyceraldehyde-3-phosphate, dihydroxyacetone phosphate and succinic acid) in test set showed the same trend as those in training set, although did not reach statistical significance.

These results demonstrated the glucose metabolic signature of schizophrenia subjects in PBMCs was significantly distinguishable from that of non-schizophrenia subjects.

### A potential diagnostic biomarker for schizophrenia

Although unique glucose metabolic pattern provided great potential for diagnosis of schizophrenia, measurements of all glucose metabolites were not convenient and economical in clinical. To identify a simplified diagnostic biomarker for schizophrenia, a step-wise optimization algorithm based on AIC was applied. The statistical analysis demonstrated that ribose 5-phosphate which involved in pentose phosphate pathway showed the highest diagnostic performance in identifying first-episode drug-naïve schizophrenia subjects in training set. The area under ROC curve (AUC) was 1.00 in discriminating schizophrenia subjects from healthy controls or major depression subjects in training set. As an AUC of 1 indicates perfect discrimination, these results demonstrated that ribose 5-phosphate in PBMCs may be a potential biomarker for first-episode drug-naïve schizophrenia subjects.

We found that ribose 5-phosphate was consistently increased in unmedicated schizophrenia subjects relative to healthy controls in both training set and test set. However, levels of ribose 5-phosphate in medicated schizophrenia subjects was significantly lower than healthy controls in test set (Additional file 1: Table S2). This findings indicated that antipsychotics could reduce the diagnostic performance of ribose 5-phosphate for schizophrenia.

## Discussion

Schizophrenia is a widespread and debilitating mental disorder. Current diagnosis of schizophrenia remains subjective, and the underlying molecular mechanisms remains largely unknown. Here, we found that majority of glucose metabolites in PBMCs were disturbed in schizophrenia subjects, which was in contrast to the findings of depression subjects. Furthermore, ribose 5-phosphate showed the highest diagnostic performance for first-episode drug-naïve schizophrenia subjects among the identified glucose metabolites. These results suggest that disturbance of glucose metabolism pathway may be implicated in the onset of schizophrenia, and may have utility in diagnosis of schizophrenia.

Antipsychotic medication and demographic factors (such as age, sex, obesity) were considered as possible confounders affecting the glucose metabolism [32, 33]. We circumvented this problem by carrying out analyses of PBMCs samples from first-episode drug-naïve schizophrenia subjects and demographic-matched controls. Hence, in this study, the glucose metabolism pattern of schizophrenia subjects might truly reflect the pathophysiologic changes inherent in the schizophrenia disease state. Consistent with these findings, several studies have also revealed poor glucose regulation and abnormal expressions of glucose metabolism related molecules in unmedicated schizophrenia subjects [12, 18, 20]. Different from previous studies, we found that antipsychotics had only a subtle effect on glucose metabolites in PBMCs except ribose 5-phosphate in this study. The discrepancy with our results may be due to the more stable microenvironment in PBMCs than peripheral body fluids and antipsychotic drug-sensitive tissues.

Glucose is mainly metabolized via the glycolytic pathway and TCA cycle to provide energy and biosynthetic intermediates. Glucose metabolism homeostasis is fundamental to maintain normal brain functions. In this study, of the 12 metabolites relevant to glycolysis and TCA cycle, 50% metabolites were increased while only 33.3% metabolites were decreased in first-episode drug-naïve schizophrenia subjects compared to healthy controls. In concordance with this finding, a recent proteomics study reported that majority (87.5%) of glucose metabolic enzymes in PBMCs were significantly elevated in first-episode drug-naïve schizophrenia subjects relative to healthy controls [18]. Besides, several glucose metabolic enzymes such as aldolase C, triosephosphate isomerase, glyceraldehydes-3-phosphate dehydrogenase, phosphoglycerate kinase 1, phosphoglycerate mutase were consistently elevated in both brain tissues and PBMCs of schizophrenia as described in previous studies [16, 34–37]. Combined with these evidences, we deduced that glycolysis pathway and TCA cycle may be activated in the early stage of schizophrenia onset. Other groups have also reported higher levels of ATP and increased gene/protein expression associated with glucose metabolism in schizophrenia patients [16, 38, 39], further supporting our findings.

Pentose phosphate pathway is another important branch of glucose metabolism that functions in the formation of NADPH for biosynthetic processes and cellular redox balance. Ribose 5-phosphate is the main product of this pathway. Here, ribose 5-phosphate was increased in PBMCs of first-episode drug-naïve schizophrenia subjects compared to healthy controls, suggesting disturbance of pentose phosphate pathway may play an important role in the onset of schizophrenia. In agreement with this speculation, previous studies have also reported increased expressions of pentose phosphate pathway related molecules in schizophrenia [36, 40].

As disturbances of glucose metabolism have also been observed in other psychiatry disorders, such as major depression. The question then arises whether disturbance of glucose metabolism observed in this study reflects schizophrenia-specific pathophysiological changes. To elucidate this question, we also recruited first-episode drug-naive major depression subjects that shared a number of factors in common with schizophrenia subjects. We found that only few kinds of glucose metabolites were differentially expressed in major depression subjects compared to healthy controls, reflecting subtle alterations of glucose metabolism pathways in this disorder. The results contrast with those of previous studies that reported significant disturbances of glucose metabolism in major depression subjects [41, 42]. The discrepancy may be caused by the differences in sample type, study population, antidepressant treatment and detected components. Here, we found that majority of glucose metabolites were distinguishable between major depression and schizophrenia subjects. The results reflected that distinct, rather than generalized, glucose metabolic changes exist in PBMCs of schizophrenia.

Certain limitations of this study should be noted. Firstly, the sample size of first-episode drug-naïve schizophrenia subjects used in this study was relatively small. This was limited by the fact that large clinical centers (~1,500 patients) recruit only 10–20 antipsychotic-naive patients who are free of substance abuse or comorbidities per year. Further studies with larger cohorts should be performed to validate these findings, especially with regard to the diagnostic performance of ribose 5-phosphate in schizophrenia. Secondly, all subjects were of the same ethnicity and were recruited from the same site. Thus, ethno- and site-specific biases cannot be ruled out. Further studies involving heterogeneous populations from multiple clinical sites are required.

## Conclusion

In this study, we provide evidence that severe disturbance of glucose metabolism in PBMCs may be implicated in the onset of schizophrenia. Moreover, ribose 5-phosphate showed a high diagnostic performance for first-episode drug-naïve schizophrenia subjects. These findings will be valuable in uncovering the pathogenesis of schizophrenia and facilitate to develop diagnostic tools for this disorder.

## Additional file

Additional file 1. The characteristics of glucose metabolites and its relation to antipsychotic use.
