# Supplementary material for: Severe disturbance of glucose metabolism in peripheral blood mononuclear cells of schizophrenia patients: a targeted metabolomic study
Source: J Transl Med. 2015 Jul 14;13:226. doi: 10.1186/s12967-015-0540-y (PMC4501123; doi:10.1186/s12967-015-0540-y)
Supplement: Additional file 1. — The characteristics of glucose metabolites and its relation to antipsychotic use. [file 12967_2015_540_MOESM1_ESM.docx]

**Supplemental Table 1. The characteristic ions and retention times of metabolites**

| **Metabolites** | **Retention time (min)** | **Quantitative ion (m/z)** | **Qualitative ion (m/z)** |
| --- | --- | --- | --- |
| Glucose | 16.17 | 319 | 205; 160 |
| Glucose 6-phosphate | 20.09 | 387 | 357; 471 |
| Fructose 6-phosphate | 19.99 | 357 | 315; 387 |
| Fructose | 15.87 | 307 | 103; 217 |
| Glyceraldehyde-3-phosphate | 14.14 | 160 | 328; 211 |
| Dihydroxyacetone phosphate | 14.55 | 400 | 315; 299 |
| Glycerol 3-phosphate | 14.63 | 357 | 315; 445 |
| Glycerate 3-phosphate | 15.10 | 459 | 357; 387 |
| Pyruvate | 5.48 | 174 | 115; 89 |
| Lactic acid | 5.64 | 117 | 191 |
| Citric acid | 15.21 | 273 | 363; 375 |
| Succinic acid | 9.02 | 129 | 247 |
| Ribose 5-phosphate | 18.19 | 315 | 357; 403 |

**Supplemental Table 2. The comparison of glucose metabolites among healthy controls, unmedicated and medicated schizophrenia subjects in test set**

| **Metabolites** | **Metabolic pathway** | **Unmedicated SZ**  **(*vs.* Medicated SZ)** | | **Unmedicated SZ**  **(*vs.* HC)** | | **Medicated SZ**  **(*vs.* HC)** | |
| --- | --- | --- | --- | --- | --- | --- | --- |
|  |  | **Log_2_(FC) ^a^** | ***P*-value^d^** | **Log_2_(FC) ^b^** | ***P*-value^d^** | **Log_2_(FC) ^c^** | ***P*-value^d^** |
| Glucose | Glycosis | -0.22 | 1.000 | 0.51 | 0.337 | 0.73 | **0.003** |
| Glucose 6-phosphate | Glycosis | -0.39 | 0.434 | 1.19 | 0.083 | 1.58 | **0.000** |
| Fructose 6-phosphate | Glycosis | -0.27 | 1.000 | 0.86 | 0.218 | 1.13 | **0.002** |
| Fructose | Glycosis | 0.05 | 1.000 | 0.52 | 0.095 | 0.47 | **0.038** |
| Glyceraldehyde-3-phosphate | Glycosis | 0.08 | 1.000 | -1.00 | 0.060 | -1.08 | **0.005** |
| Dihydroxyacetone phosphate | Glycosis | 1.03 | 0.829 | -0.69 | 0.251 | -1.71 | **0.001** |
| Glycerol 3-phosphate | Glycosis | 0.18 | 1.000 | -0.37 | 0.194 | -0.55 | **0.004** |
| Glycerate 3-phosphate | Glycosis | 0.24 | 1.000 | 0.44 | 0.384 | 0.20 | 1.000 |
| Pyruvate | Glycosis | -0.31 | 1.000 | 0.59 | 0.798 | 0.91 | **0.028** |
| Lactic acid | Glycosis | 0.18 | 1.000 | -0.67 | 0.180 | -0.85 | **0.013** |
| Citric acid | TCA | 0.12 | 1.000 | -0.63 | 0.085 | -0.75 | **0.004** |
| Succinic acid | TCA | 0.29 | 0.382 | 0.96 | **0.001** | 0.67 | **0.010** |
| Ribose 5-phosphate | Pentose phosphate pathway | 1.71 | **0.000** | 0.47 | **0.003** | -1.25 | **0.000** |

^a^A negative log_2_(FC) indicated significantly lower expression in unmedicated schizophrenia subjects compared to medicated schizophrenia subjects;

^b^A negative log_2_(FC) indicated significantly lower expression in unmedicated schizophrenia subjects compared to healthy controls;

^c^A negative log_2_(FC) indicated significantly lower expression in medicated schizophrenia subjects compared to healthy controls;

^d^These data were analyzed using one-way ANOVA followed by Bonferroni post hoc test between two experimental groups.

Values given in bold denote statistically significant results (*P＜*0.05).

Abbreviations: SZ, schizophrenia; HC, healthy controls; FC, Fold change; TCA, Tricarboxylic acid cycle.
